# NEPdb: A Database of T-Cell Experimentally-Validated Neoantigens and Pan-Cancer Predicted Neoepitopes for Cancer Immunotherapy

**DOI:** 10.3389/fimmu.2021.644637

**Published:** 2021-04-13

**Authors:** Jiaqi Xia, Peng Bai, Weiliang Fan, Qiming Li, Yongzheng Li, Dehe Wang, Lei Yin, Yu Zhou

**Affiliations:** ^1^ State Key Laboratory of Virology, Hubei Key Laboratory of Cell Homeostasis, College of Life Sciences, Wuhan University, Wuhan, China; ^2^ Frontier Science Center for Immunology and Metabolism, Wuhan University, Wuhan, China

**Keywords:** neoantigen, HLA, cancer immunotherapy, somatic mutation, neoepitope, MHC, T-cell

## Abstract

T-cell recognition of somatic mutation-derived cancer neoepitopes can lead to tumor regression. Due to the difficulty to identify effective neoepitopes, constructing a database for sharing experimentally validated cancer neoantigens will be beneficial to precise cancer immunotherapy. Meanwhile, the routine neoepitope prediction *in silico* is important but laborious for clinical use. Here we present NEPdb, a database that contains more than 17,000 validated human immunogenic neoantigens and ineffective neoepitopes within human leukocyte antigens (HLAs) *via* curating published literature with our semi-automatic pipeline. Furthermore, NEPdb also provides pan-cancer level predicted HLA-I neoepitopes derived from 16,745 shared cancer somatic mutations, using state-of-the-art predictors. With a well-designed search engine and visualization modes, this database would enhance the efficiency of neoantigen-based cancer studies and treatments. NEPdb is freely available at http://nep.whu.edu.cn/.

## Introduction

Immunotherapy has clinical activity in cancers by targeting cancer antigens (1). To understand and develop tumor-specific immunity for cancer therapy, many studies have been taken over recent years to identify cancer antigens that can be recognized by immune cells, especially T-cells (2). Usually, these antigens can be naturally processed and presented on cancer cells by human leukocyte antigens (HLAs) and recognized by T-cells. Upon recognition of epitopes (peptide-MHC complex), T-cells can be activated to proliferate and destroy cancer cells.

There are two broad categories of cancer antigens: self- and nonself-antigens (3). Self-antigens, which represent molecules (peptides, lipids, etc.) derived from cancerous cells but seldom be found in normal cells (4). Even though many clinical trials have been done by targeting cancer self-antigens, limited effectiveness was shown during these studies. This phenomenon may be partially explained by immune central tolerance, which eliminates the high-avidity T-cells directly against these self-antigens. Meanwhile, therapies targeting self-antigens may also increase the risks of self-destruction of normal tissues or cross-reactivity against unanticipated targets expressed in normal tissues. For these reasons, it is needed to find safe and effective cancer antigens for clinical trials. Nonself-antigens include cancer-specific peptides that arise from non-synonymous mutations in cancer genomes (called neoantigens) and viral peptides expressed in virus-induced cancers. In contrast to self-antigens, mutation-associated neoantigens are foreign to the immune system and may induce strong and safe antitumor responses (5). Conceptually, neoantigens are not present in normal tissues, hence the immune system is not tolerant to them and recognizes them as foreign antigens by T-cell receptors (TCRs) (6, 7). Recent studies have demonstrated that neoantigen-reactive T-cells targeting can mediate substantial objective clinical regressions in patients with metastatic solid tumors (8, 9). Furthermore, neoantigen-reactive T-cells are proved to be important mediators of immune checkpoint blockade, cancer vaccination, and adoptive T-cell transfer (ACT) (10).

With the rapid development of neoantigen-based immunotherapies, the number of neoantigen has increased drastically over the past few years. Currently, neoantigen-related data, including antigen sequence, antigen expression level, protein carcinogenesis, MHC presentation, clinical activity, and corresponding TCR sequence, are sparse, non-unified, difficult to retrieve, and hard to analyze. To overcome these issues, recent studies have curated cancer antigen data and constructed several publicly available cancer antigen resources. For example, the Cancer Antigenic Peptide Database contains cancer self-antigen and nonself-antigens, but it has only 65 entries for cancer neoepitopes. The TANTIGEN database contains many types of cancer antigens, however, it lacks ineffective neopeptide-HLA data and pan-cancer predicted neoepitopes (11). TSNAdb (Tumor-specific Neoepitopes database) contains predicted neoepitopes without experimental validation information (12). The NeoPeptide database contains neoepitopes with nucleic acid mutation, amino acid mutation, and relevant MHC curated from literature. However, it is hard to know which entry is truly immunogenic *in vivo*, which limits potential applications (13).

To fill these gaps, we constructed a cancer NeoEPitope database (NEPdb) by curating experimentally validated neoepitopes and ineffective neopeptides from literature using a semi-automatic pipeline. First, this database contains 17,549 experimentally validated neoepitopes and ineffective neopeptides with associated information including corresponding protein sequences, mutant positions, HLA (both class I and II) alleles, T-cell activation information, TCR sequences, and clinical outcome in human cancers, here termed the Validated Neopeptide Dataset (VND). Second, we comprehensively identified shared HLA-I neoepitope landscape at the human pan-cancer level using cancer somatic mutation data from COSMIC (Catalogue Of Somatic Mutations In Cancer) by state-of-the-art predictors NetMHCpan 4.0 and HLAthena (14–16). We screened 16,745 non-synonymous mutations from 683 COSMIC-curated cancer genes and predicted the binding potentials between 516,036 mutant peptides (8, 9, 10, and 11-mers) and 95 common HLA-I alleles. All these data were recorded in the Predicted Neopeptide Dataset (PND). Furthermore, we developed an efficient search engine and well-designed visualization technology in the NEPdb web server to present the above data. We hope that NEPdb can serve as a timely and valuable resource to facilitate the study of neoantigen immunogenicity and the application of clinical cancer immunotherapy. NEPdb is freely available at http://nep.whu.edu.cn/.

## Materials and Methods

### Data Collection

The data of VND were curated from published literature in a semi-automatic manner. Herein, the mutant peptide proved to be immunogenic is termed ‘neoantigen’; the mutant peptide with validated immunogenicity in the context of a certain HLA is termed ‘neoepitope’; and the mutant peptide with uncertain immunogenicity is termed ‘neopeptide’. We programmatically screened related literature by parsing and filtering abstracts with natural language processing toolkit in using multiple specific keywords, such as ‘cancer immunotherapy’, ‘neoepitope’, ‘neoantigen’, ‘immunogenicity’, ‘mutation’, ‘HLA’, etc. All literature results were further manually filtered to keep those containing neoantigen-related data with explicit experimental validation for further curation (**Supplementary Figure S1**).

In PND, cancer-related genes were collected from the Cancer Gene Census project (https://cancer.sanger.ac.uk/census) in COSMIC. The Cancer Gene Census (CGC) database includes 683 catalogued genes with many mutations that have been causally implicated in cancers. From those genes, we collected 16,745 non-synonymous mutations that occur frequently (at least three times) in COSMIC for further analyses.

### Data Curation and Validation of VND

In VND, major efforts were made to ensure the data reliability in our database. All screened papers were further manually read and curated. All of the peptides must be explicitly defined with experimental validation in the original references. The data integrity was evaluated and the experimental condition of each neoepitope or neopeptide was recorded in a unified format by immunology specialist curators.

The original data curated from literature were further standardized and corrected in a customized format. Original gene names (or transcript IDs) came from three databases: NCBI (https://www.ncbi.nlm.nih.gov/), ENSEMBL (http://grch37.ensembl.org), and UCSC (http://genome.ucsc.edu/). To make our database more convenient to search, we unified gene names as the generic NCBI gene symbol. To facilitate users to search the relevant literature, the details of references (such as PMID, journal, published date, and reference title) were added to the database. Next, we verified or corrected the mutation information (**Supplementary Figure S1**). Firstly, the mutation position of each neoepitope or neopeptide was checked, and the entry was removed for the cases that the mutation position in the literature is different from the position of the wild-type amino acid in the sequence from the NCBI database. Secondly, for those peptides occurring multiple times in their full-length protein sequences, the occurrence rank numbers were recorded in NEPdb. Thirdly, when the information of either wild-type peptide, mutant peptide, or mutation position was missing, it was inferred from the full-length protein sequence.

Curated neoantigens are classified into two major categories: 1) peptides tested *in vitro* or *in vivo* reported to elicit T-cell response or clinical response, marked with ‘P’ as positive (immunogenic); 2) peptides measured *in vitro* or *in vivo* but reported not to elicit T-cell response or clinical response, marked with ‘N’ as negative (ineffective). Each antigen was recorded in NEPdb as an individual entry. Entries were curated and characterized according to the following fields on experimental conditions: tumor type, gene symbol of peptide, peptide sequence of both wild type and mutant, corresponding HLA allele, assay details (T-cell source, APC source, antigen type), clinical trial details, reference details, and antigen validation with computational methods.

### Neoepitope Prediction of PND

In Predicted Neopeptide Dataset (PND), cancer genes and non-synonymous mutations were selected based on the occurrence frequency in COSMIC. Next, a neopeptide pool was computationally created for each cancer gene derived non-synonymous mutations associated peptides potentially binding to HLA-I. For each mutation, we performed a comprehensive assessment of peptides 8–11 amino acids in length at every position surrounding a somatic mutation. That is, each missense mutation has a neopeptide pool composing of 8-, 9-, 10-, 11-mer peptides with the corresponding amino acid change in their sequences. Thus, a non-synonymous mutation could generate up to 38 peptides, for which we predicted the binding potentials with 95 HLA class I alleles using NetMHCpan 4.0 (16) and HLAthena (15), respectively. The collection of 95 alleles (31 HLA-A, 40 HLA-B, 21 HLA-C, and 3 HLA-G) is known to cover at least one allele in 95% of individuals worldwide (15). We extracted and recorded the prediction results (rank% and affinity (IC50) from NetMHCpan4.0, MSi and prank.MSi from HLAthena). The prediction results are displayed in the form of dynamic heatmaps, and users can visualize the corresponding combination of peptide and HLA using adjustable thresholds.

### Usage Notes

We intend to continuously update NEPdb by collecting more cancer neoepitopes and non-immunogenic data in VND. The curation rules will be constantly re-evaluated for new experimental techniques. To enhance the efficiency of collecting literature with neoantigen data, we have trained an SVM model based on extracting TF-IDF features (17) from the abstracts and full texts of positive literature containing neoantigen data versus negative literature related to cancer and T-cell but without neoantigen data. We also encourage researchers to share neoantigen-related data through our database. It is easy to upload the data in a standard format shown on the submission page or send us related references by E-mail.

The curated data in the database are also provided as CSV files for download, which can be opened in Excel or any text editor, and directly used in downstream analysis.

NEPdb provides friendly help documentation on using the database. In the glossary page, all the abbreviations in the database are described or explained in detail.

## Results

### Overview of NEPdb

**Figure 1:**
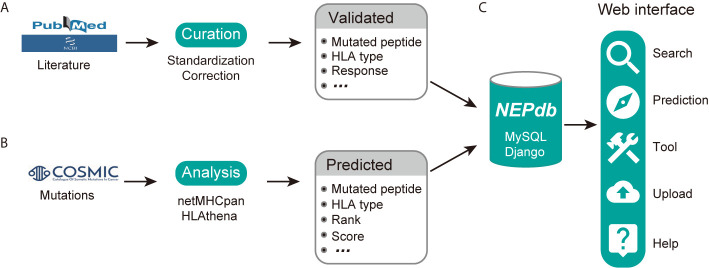
NEPdb content and construction. **(A)** Manual collection and curation of experimentally validated neoepitope and non-immunogenic peptides information. **(B)** Computational analysis of HLA-peptides for pan-cancer gene mutations. **(C)** Database implementation and overview of the web interface.

NEPdb is a database of experimentally validated immunogenic neoepitopes and ineffective neopeptides, and computationally predicted pan-cancer HLA-neopeptides, which are of great importance for vaccines and immunotherapy for human cancers (**Figures 1A, B**). These data can be queried and visualized with a friendly web interface (**Figure 1C**).

**Figure 2:**
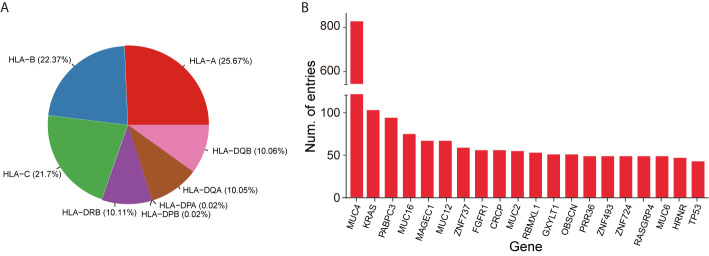
Statistics of experimentally validated data in NEPdb. **(A)** The distribution of HLA alleles. **(B)** Top 20 genes with the most validated neoepitope and non-immunogenic peptides in NEPdb.

We parsed existing literature and gathered validated neoepitopes and ineffective peptides with a semi-automatic pipeline. The statistics of validated HLA-peptides included in this database are shown in **Table 1**. Currently, the dataset termed the Validated Neopeptide Dataset (VND), encompassed 173 neoepitopes and 17,376 ineffective neopeptides of human cancers from 41 published literature in recent years. Most of them were tested with T-cell assays *in vitro*, or clinical vaccine immunizing, or T cell based adoptive transfer *in vivo*. Each entry includes cancer type, HLA allele type (I and II), gene symbol, peptide sequence, assay details, TCR sequences, and other characteristics, which are important in cancer immunotherapy. Considering that some tumor types contain high mutational burden, it is explicable that nearly 80% of entries come from melanoma and non-small cell lung cancer for now. The immunogenic dataset also contains TCR information (variable region, diversity region (heavy chain only), joining region, and complementarity-determining 3 region CDR3), if available in literature. The distribution of HLA alleles and the top 20 genes with the largest number of entries are shown in **Figures 2A, B**, respectively. The VND dataset was recorded in the “SEARCH” section.

**Table 1:** Data statistics in NEPdb.

Data content	HLA-I data statistics^a^	HLA-II data statistics^b^	Total
Entry (Total)	12,239	5,310	17,549
Entry (Positive)^c^	155	18	173
Entry (Negative)^d^	12,084	5,292	17,376
Tumor type	22	11	23
HLA Allele	60	35	95
Gene	2,063	811	2,068
Protein sequence	2,332	895	2,337

^a^Number of peptides which bind to HLA-I alleles. ^b^Number of peptides which bind to HLA-II alleles. ^c^Immunogenic neoepitope entries. ^d^Ineffective neopeptide-HLA entries.

Cancers with dominant mutations can often be effectively treated by targeting the mutation-associated antigens. For pan-cancer neoepitope prediction, 16,745 dominant non-synonymous mutations were selected from 683 cancer genes (Cancer Gene Census) and occurred at least 3 times leading to amino acid changes from COSMIC. For each dominant non-synonymous mutation, we generated neopeptides in the range of 8-11 mers (the possible peptide-HLA-I binding lengths) resulting in a pool of 516,036 neopeptides. Next, we applied two state-of-the-art peptide-HLA binding prediction algorithms for neoepitope prediction. The first program is NetMHCpan 4.0, a widely used algorithm, maintained by Nielsen et al, and the other is HLAthena, a newly developed algorithm and trained by >185,000 mass spectrometry (MS)-identified peptides from cell lines (15, 16). We predicted the binding probability of each neopeptide from the pool with 95 HLA-I alleles (a total of 516,036 × 95 interactions). The HLA-I distributions among the VND and PND are presented in **Supplementary Figure S2**. To filter predicted-neopeptides by HLA-I for different human populations, users can query the Allele Frequency Net Database (AFND, http://allelefrequencies.net), which curated HLA frequency from various samples in different human populations by country, region, etc (18). The relevant mutation information and COSMIC ID are recorded in NEPdb. This dataset was called the Predicted Neopeptide Dataset (PND). The PND dataset was recorded in the “PREDICTION” section.

### Interface of VND

**Figure 3:**
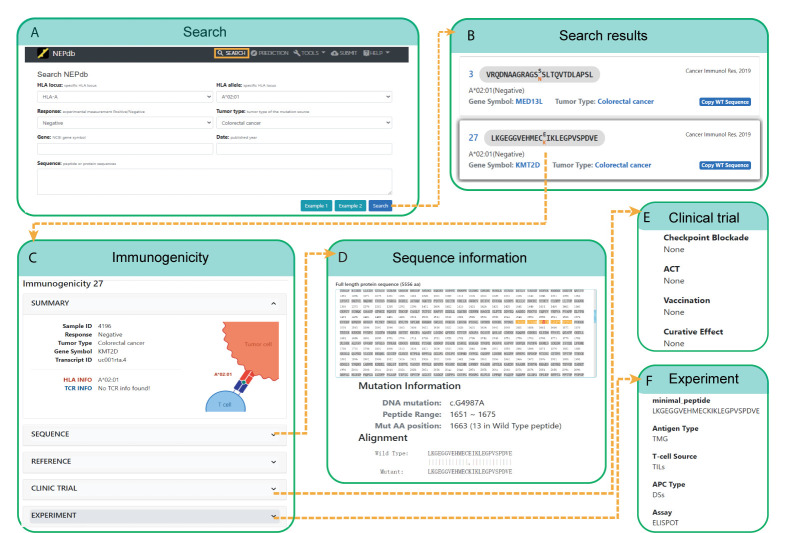
A comprehensive view of NEPdb search and query interface. **(A)** Search options include HLA locus, response, tumor type, sequence, and gene. **(B)** Query results include wild peptide, mutation peptide, mutation AA (amino acid), gene symbol, HLA type, literature, etc. **(C)** Details of a selected peptide from the results table. **(D-F)** Result pages of full protein sequence **(D)**, clinical trial **(E)**, and experimental information **(F)**.

VND (Validated Neopeptide Dataset) of NEPdb can be searched in a friendly web user interface with various query options (**Figure 3**). Single or multiplex combination filters include response status, specific peptide or protein sequence, tumor type, HLA type, gene symbol, and publication year. We have provided examples on the searching page to guide users on the search conditions and result page. The choices of HLA types, response status, and tumor types are provided in drop-down menus to simplify the query. While the mouse hovers over the search conditions, a short hint is provided for users. Users can query neopeptides using the publication year or official gene symbols within the search box. After the search query is submitted, a new result page will be generated, in which the entries can be ordered with different conditions. The detailed information about genes and cancers can be viewed through the links to GeneCards and National Cancer Institute websites, respectively. Users could set the number of entries per page. While only some important antigen features are displayed on the result page, *via* the hyperlink on the antigen sequence or ID, users can view the complete information including sample information, sequence information, experiment, reference, etc.

### Interface of PND

The “PREDICTION” page mainly displays the predicted results of PND (Predicted Neopeptide Dataset) on pan-cancer mutations. When users enter a transcript ID (ENST ID) or a gene symbol, a dynamic graphical interface will be presented, starting with a table containing basic information of the short peptides, including gene name, mutation information, etc. A dynamic scatter diagram shows the overlap of the predicted results from the two algorithms, NetMHCpan 4.0 and HLAthena (**Supplementary Figure S3A**). Two separate heatmaps display the prediction results from NetMHCpan 4.0 and HLAthena, respectively (**Supplementary Figure S3B**). Users can change the scale to customize the threshold to dynamically view the results. The detailed information about mutations can be viewed through the link to COSMIC.

### Tool Interface

The web server mainly provides two tools, similarity search and subsequence search to the validated and the predicted neopeptides in NEPdb. For similarity search, users could enter a peptide sequence and will obtain the list of neopeptides that are closely similar (>70%) to the input. For subsequence search, users could enter a peptide sequence and the tool will search neopeptides within it and highlight the hit(s). When users enter DNA or RNA sequence, the tool will first translate it into protein sequences according to the six open reading frames, and then execute the above steps on translated protein sequences.

### Application of NEPdb

For cancer immunotherapy, it is crucial to prioritize tumor neoepitopes with binding prediction algorithms. However, it is difficult to benchmark which algorithm performs better, due to the lack of gold standard datasets. The validated neoepitopes in NEPdb offer us the possibility to evaluate the performance of neoepitope prediction algorithms.

We tested 9 commonly used algorithms for peptide-HLA binding prediction, including SMM 1.0 (19), Consensus 2.18 (20), MHCflurry 1.20 (21), IEDB recommended 2.19 (20), NetMHC 4.0 (22), NetMHCpan 4.0 (16), NetMHCcons 1.1 (23), PickPocket 1.1 (24), and HLAthena (15). The evaluation results are shown in **Supplementary Figures S4A, B**. We used ‘sensitivity’ or ‘true positive rate’ to evaluate the above algorithms, which is computed by dividing the number of positive neoantigens correctly identified to the total number of positive neoantigens. In summary, based on current data in NEPdb, the top three algorithms are NetMHCcons 1.1 (90%), NetMHCpan (90%) and HLAthena (85%). It should be noted that HLAthena might achieve better performance after providing auxiliary information such as gene expression. This evaluation would be more reliable when the ground-truth dataset becomes larger, which is one of our aims to construct and continuously maintain NEPdb.

## Discussion

Cancer cells can be naturally recognized by T-cells with the cancer antigens presented by HLAs on the cell surface. The known tumor antigens are either self-epitopes derived from normal self-proteins or nonself-peptides derived from translation of somatic mutations (neoantigens). Recently, neoantigens have been validated to be able to elicit T-cell response and clinical response in patients. However, the neoantigen data and the relevant clinical information are hidden in a large number of dispersed literature and have not been structurally collected yet. Here, we have constructed NEPdb as a reference for the research community, providing experimentally or clinically validated neoantigen information. Even though some other databases have been established to show validated neoantigens, our NEPdb have integrated T-cell assay details, TCR information, clinical outcome, and a variety of other useful features, and thus can provide a systemic overview of immune events in neoantigen-based cancer immunotherapy. Moreover, we have also integrated HLA-II neoantigens and ineffective neopeptide data for neoantigen immunological study. Finally, we have provided a friendly interface to access the immunogenic neoepitopes and ineffective neopeptides from cancer immunotherapy studies.

Given that personalized neoantigen predictions might become routine in further neoantigen-based cancer immunotherapy (25), we have generated a dataset that contains pan-cancer level HLA-I neoepitope prediction based on the widely used algorithm NetMHCpan 4.0 and the high-performance algorithm HLAthena. We plan to integrate predicted HLA-II neoepitopes (important for CD4+ T-cell antitumor responses) into our database when HLA-II binder prediction tools achieve better accuracy. Currently, it is still very challenging to predict immunogenic neoantigens and to develop an effective scoring metric for neoantigen prioritization. Continuously collecting experimentally validated neoantigens by incorporating data-mining tools in literature curation are helpful for optimizing neoantigen prediction algorithms. Meanwhile, it will be very valuable to develop high-throughput techniques for identifying immunogenic neoantigens.

Overall, we believe that NEPdb is a valuable resource and can lighten the burden of neoantigen prediction for immunologists and clinicians.

## Data Availability Statement

The original contributions presented in the study are included in the article/**Supplementary Material**. The data processing scripts of our pipeline have been deposited in GitHub (https://github.com/zhouyulab/nepdb). Further inquiries can be directed to the corresponding authors.

## Author Contributions

YZ, LY and PB conceived the study. PB and YL collected the literature and parsed the data. JX, PB, WF, QL and YL performed the analysis. JX, WF and DW constructed the database and web interface. PB, JX, YZ and LY wrote the manuscript. All authors contributed to the article and approved the submitted version.

## Funding

This research was supported by National Natural Science Foundation of China (31922039 to YZ; 31870728 and 31470738 to LY) and the Fundamental Research Funds for the Central Universities (2042020kf1069 to YZ; 2042020kfxg02 to LY).

## Conflict of Interest

The authors declare that the research was conducted in the absence of any commercial or financial relationships that could be construed as a potential conflict of interest.

## Supplementary Material

Supplementary Figure 1 Semi-automatic pipeline for curating neoantigens from literature.

Supplementary Figure 2 Comparison of HLA distribution and peptide number for VND and PND. The number of peptides corresponding to different HLA-I alleles are shown for the immunogenic neoantigen dataset (VND), the ineffective neoantigen dataset (VND), the HLAthena-predicted neoantigen dataset (PND), and the NetMHCpan-predicted neoantigen dataset (PND), respectively.

Supplementary Figure 3 Data visualization of predicted HLA-peptides for pan-cancer gene mutations. **(A)** Dynamic scatter plot of predicted HLA-peptides (netMHCpan rank vs. HLAthena rank in log_10_ scale). The rank threshold can be adjusted to select reliable HLA-peptides highlighted in blue towards the lower left corner. **(B)** Heatmap showing the predicted bindings between peptides (left) and 95 HLA-A, -B, -C, and –G alleles (bottom) by NetMHCpan. The blue square indicates the HLA-peptides with rank less than 2.

Supplementary Figure 4 Performance of nine HLA class I prediction algorithms. **(A)** Overall performance of 9 algorithms evaluated on the experimentally validated immunogenic data in NEPdb. Predicted binders and non-binders are shown in green and red, respectively. Each algorithm has its own coverage of HLA alleles. **(B)** The true positive rates in percentage for the 9 algorithms as in A.
