# Supplementary figures and images for: NEPdb: A Database of T-Cell Experimentally-Validated Neoantigens and Pan-Cancer Predicted Neoepitopes for Cancer Immunotherapy
Source: Front Immunol. 2021 Apr 13;12:644637. doi: 10.3389/fimmu.2021.644637 (PMC8078594; doi:10.3389/fimmu.2021.644637)

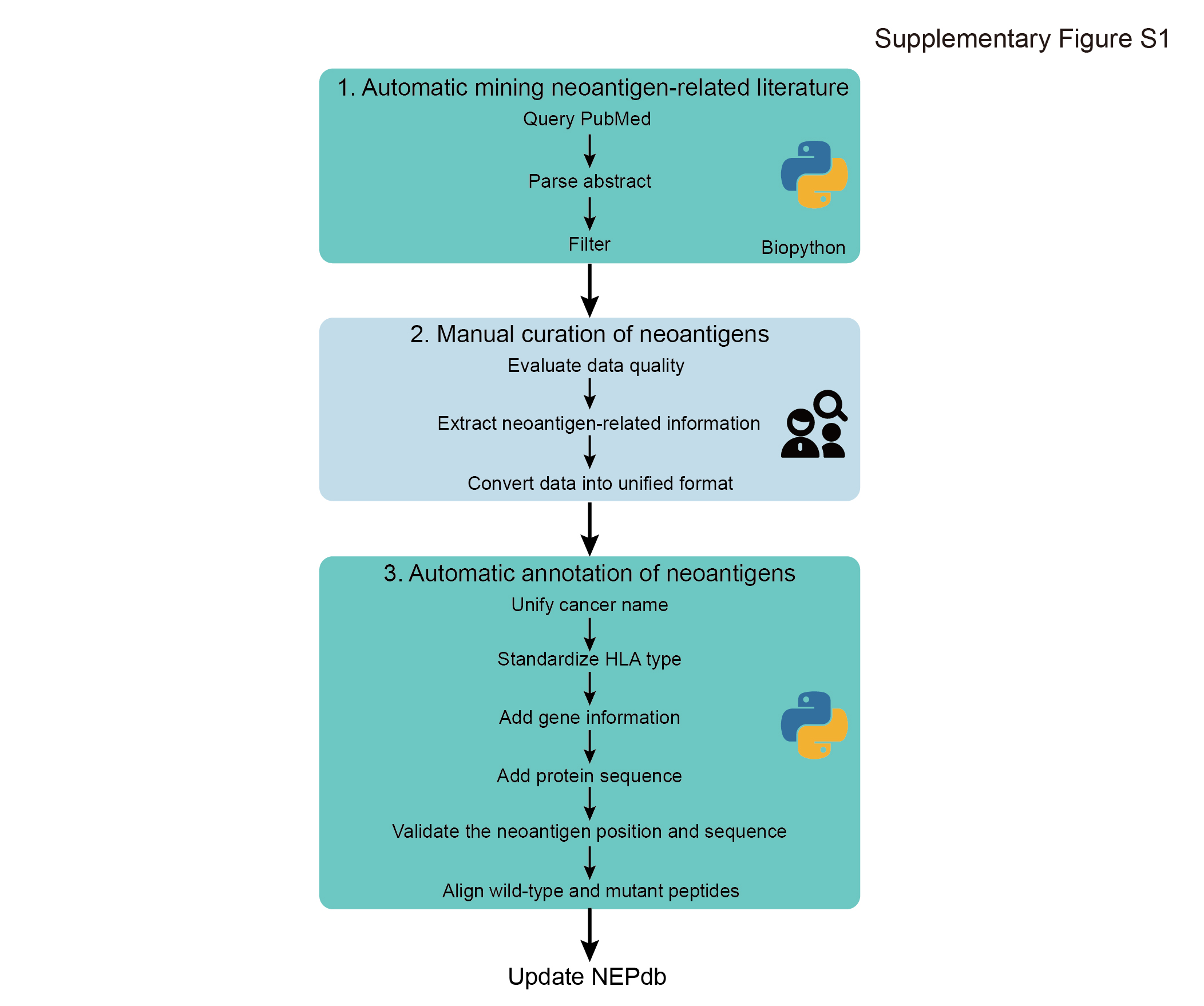

Supplement: Supplementary Figure 1 — Semi-automatic pipeline for curating neoantigens from literature. [file Image_1.jpeg]

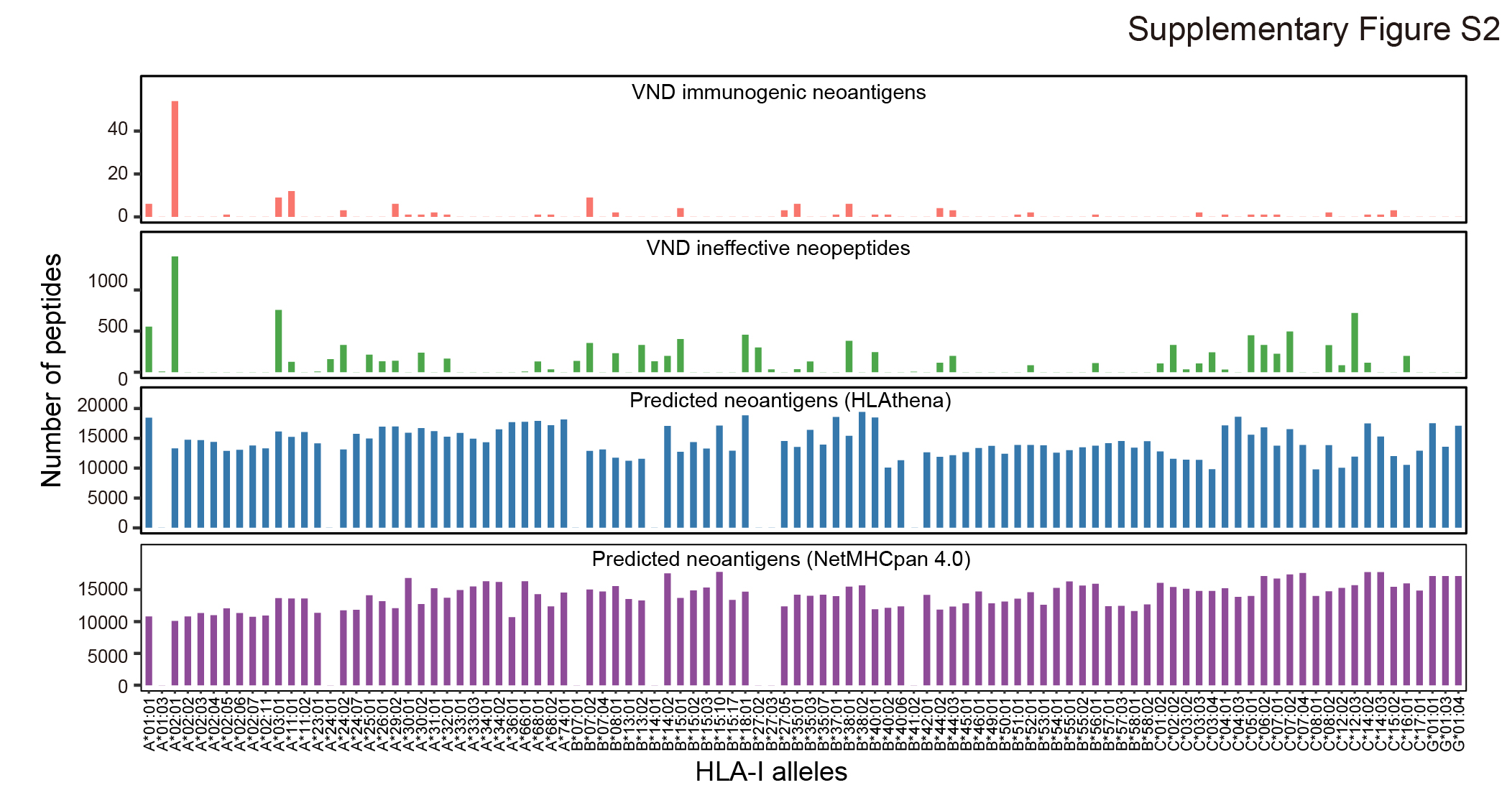

Supplement: Supplementary Figure 2 — Comparison of HLA distribution and peptide number for VND and PND. The number of peptides corresponding to different HLA-I alleles are shown for the immunogenic neoantigen dataset (VND), the ineffective neoantigen dataset (VND), the HLAthena-predicted neoantigen dataset (PND), and the NetMHCpan-predicted neoantigen dataset (PND), respectively. [file Image_2.jpeg]

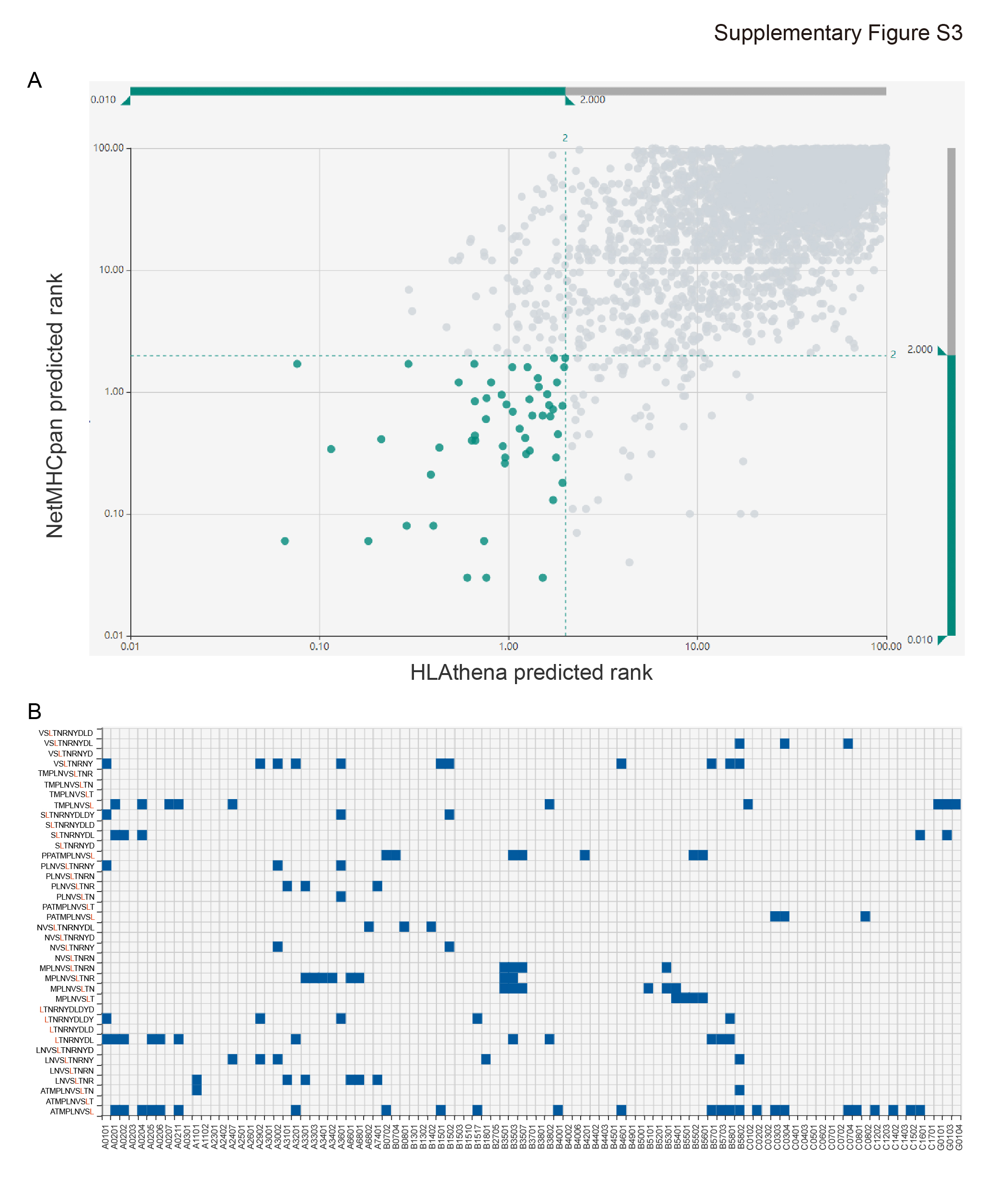

Supplement: Supplementary Figure 3 — Data visualization of predicted HLA-peptides for pan-cancer gene mutations. (A) Dynamic scatter plot of predicted HLA-peptides (netMHCpan rank vs. HLAthena rank in log10 scale). The rank threshold can be adjusted to select reliable HLA-peptides highlighted in blue towards the lower left corner. (B) Heatmap showing the predicted bindings between peptides (left) and 95 HLA-A, -B, -C, and –G alleles (bottom) by NetMHCpan. The blue square indicates the HLA-peptides with rank less than 2. [file Image_3.jpeg]

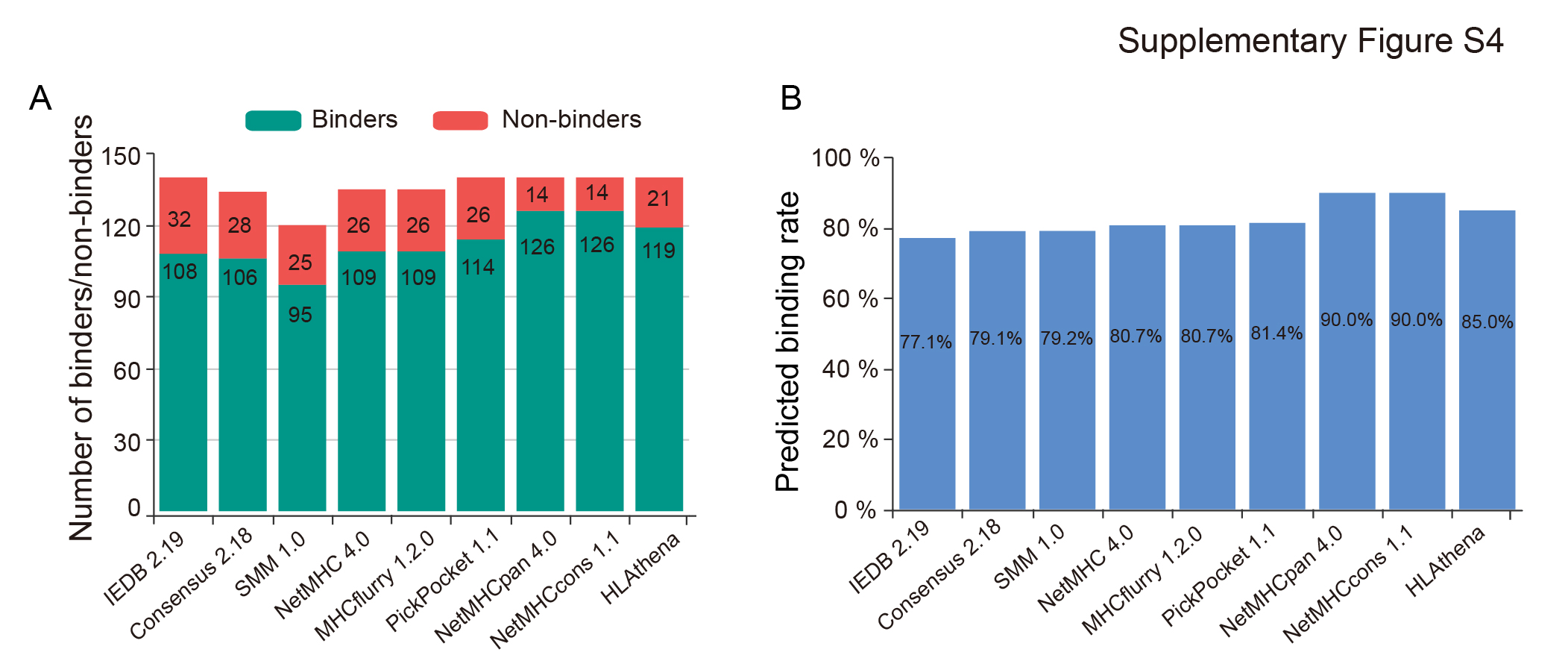

Supplement: Supplementary Figure 4 — Performance of nine HLA class I prediction algorithms. (A) Overall performance of 9 algorithms evaluated on the experimentally validated immunogenic data in NEPdb. Predicted binders and non-binders are shown in green and red, respectively. Each algorithm has its own coverage of HLA alleles. (B) The true positive rates in percentage for the 9 algorithms as in A. [file Image_4.jpeg]
